# Chromosome stability of synthetic *Triticum turgidum–Aegilops umbellulata* hybrids

**DOI:** 10.1186/s12870-024-05110-8

**Published:** 2024-05-13

**Authors:** Zhongping Song, Yuanyuan Zuo, Wenjia Li, Shoufen Dai, Gang Liu, Zongjun Pu, Zehong Yan

**Affiliations:** 1 Triticeae Research Institute, Sichuan Agricultural University, Chengdu, 611130 P. R. China; 2 Crop Research Institute, Sichuan Academy of Agricultural Science, Chengdu, 610066 P. R. China; 3 Environment-friendly Crop Germplasm Innovation and Genetic Improvement Key Laboratory of Sichuan Province, Chengdu, 610066 P. R. China; 4 Neijiang Normal University, Neijiang, 641000 P. R. China

**Keywords:** Unreduced gametes, *Turgidum turgidum*–*Aegilops umbellulata*, Variation in chromosome number and structure, Chromosome loss and gain, Molecular cytogenetics

## Abstract

**Background:**

Unreduced gamete formation during meiosis plays a critical role in natural polyploidization. However, the unreduced gamete formation mechanisms in *Triticum turgidum–Aegilops umbellulata* triploid F_1_ hybrid crosses and the chromsome numbers and compostions in *T. turgidum–Ae. umbellulata* F_2_ still not known.

**Results:**

In this study, 11 *T.turgidum–Ae. umbellulata* triploid F_1_ hybrid crosses were produced by distant hybridization. All of the triploid F_1_ hybrids had 21 chromosomes and two basic pathways of meiotic restitution, namely first-division restitution (FDR) and single-division meiosis (SDM). Only FDR was found in six of the 11 crosses, while both FDR and SDM occurred in the remaining five crosses. The chromosome numbers in the 127 selfed F_2_ seeds from the triploid F_1_ hybrid plants of 10 crosses (no F_2_ seeds for STU 16) varied from 35 to 43, and the proportions of euploid and aneuploid F_2_ plants were 49.61% and 50.39%, respectively. In the aneuploid F_2_ plants, the frequency of chromosome loss/gain varied among genomes. The chromosome loss of the U genome was the highest (26.77%) among the three genomes, followed by that of the B (22.83%) and A (11.81%) genomes, and the chromosome gain for the A, B, and U genomes was 3.94%, 3.94%, and 1.57%, respectively. Of the 21 chromosomes, 7U (16.54%), 5 A (3.94%), and 1B (9.45%) had the highest loss frequency among the U, A, and B genomes. In addition to chromosome loss, seven chromosomes, namely 1 A, 3 A, 5 A, 6 A, 1B, 1U, and 6U, were gained in the aneuploids.

**Conclusion:**

In the aneuploid F_2_ plants, the frequency of chromosome loss/gain varied among genomes, chromsomes, and crosses. In addition to variations in chromosome numbers, three types of chromosome translocations including 3UL·2AS, 6UL·1AL, and 4US·6AL were identified in the F_2_ plants. Furthermore, polymorphic fluorescence in situ hybridization karyotypes for all the U chromosomes were also identified in the F_2_ plants when compared with the *Ae. umbellulata* parents. These results provide useful information for our understanding the naturally occurred *T. turgidum–Ae. umbellulata* amphidiploids.

**Supplementary Information:**

The online version contains supplementary material available at 10.1186/s12870-024-05110-8.

## Introduction

*Aegilops umbellulata* (2*n* = 2*x* = 14, UU), as a wild relative of wheat, is a rich gene reservoir for the genetic improvement of wheat in several aspects [1, 2]. It possesses genes of resistance to biotic stresses, including stripe rust and leaf rust [3, 4], and abiotic stresses such as drought and salt tolerance [5]. For example, the leaf rust and stripe rust resistance genes *Lr76* and *Yr70* of *Ae. umbellulata* were successfully transferred into wheat [6]. These excellent genes could be introduced into wheat through direct or indirect distant hybridization, although some barriers such as hybrid sterility and abnormal pairing of chromosomes during meiosis still occur during these processes. To introduce the valuable traits/genes of *Ae. umbellulata* into common wheat via distant hybridization, it is necessary to overcome these hybridization barriers [7, 8]. Compared with direct hybridization with wild relative species, the use of amphidiploids between wheat and wild relative species as a bridge material can overcome these hybridization barriers to a certain extent. The development of amphidiploids depends on chromosome doubling, which can be done either by ionizing irradiation or clastogens induce chromosomal rearrangements, such as X-ray and colchicine, respectively, or via unreduced gametes [9].

Polyploid plants mainly occur through somatic chromosome doubling or through the union of two unreduced gamete formation. Both autopolyploids (e.g., potato) and allopolyploids (e.g., wheat) can be produced via the unreduced gamete pathway [10, 11]. The unreduced gametes can promote the formation of polyploid species and can also produce some intermediate materials for transferring heterologous genetic material. For example, the union of two unreduced gametes in the double haploid F_1_ hybrids (ABD, 2*n* = 3*x* = 21) between tetraploid wheat and *Ae. tauschii* produced synthetic wheat by spontaneous chromosome doubling (AABBDD, 2*n* = 6*x* = 42) [12]. The formation of unreduced gametes is mainly modulated by unreduced gamete genes [13]. Quantitative trait loci (QTLs) that contribute to unreduced gamete formation have been mapped on chromosomes 1 A, 3 A, 3 B, and 4 B of tetraploid wheat [14, 15]. Two types of unreduced gamete formation mechanisms, namely first*-*division restitution (FDR) and single-division meiosis (SDM), have been discovered in monocotyledonous wheat hybrids [11]. In the FDR, the first meiotic division is abnormal and generates a restitution nucleus, and the second division is normal and produces only dyads [16]. In the SDM, a single equational division of sister chromatids takes place at meiotic anaphase I generates dyads before the restitution nucleus is formed, and leads to the absence of the second meiosis [17]. It has been reported that the chromosome numbers of hybrids between tetraploid or hexaploid wheat and some *Aegilops* species could be naturally doubled to form amphidiploids through FDR and SDM, such as *T. turgidum–Ae. tauschii* [9], *T. turgidum–Ae. longissimia* [18], *T. aestivum–Ae. triuncialis* [19, 20], and *T. turgidum–Ae. comosa* hybrids [21]. The *T. turgidum–Ae. umbellulata* amphidiploids can be formed by doubling the chromosomes of haploid hybrids by colchicine treatment or unreduced gametes [7, 22–24] but the unreduced gamete formation process of the spontaneous doubling pathway and the chromosomal changes in the selfed progeny of *T. turgidum–Ae. umbellulata* hybrids are still unknown.

To study the meiosis processes as well as unreduced gamete formation in *T. turgidum–Ae. umbellulata* triploid F_1_ hybrids and to analyze the chromosomal changes in the selfed progeny after natural chromosome doubling, two tetraploid wheats, namely *T. turgidum* ssp. *durum* var. Langdon and ssp. *dicoccum* PI 94,668, were crossed with 10 *Ae. umbellulata* accessions to obtain 11 *T. turgidum–Ae. umbellulata* F_1_ hybrids. The results provide basic information for understanding unreduced gamete formation in *T. turgidum–Ae. umbellulata* hybrids and could also aid the generation of *T. turgidum–Ae. umbellulata* amphidiploids for potential use in transferring desirable traits/genes from *Ae. umbellulata* into wheat.

## Results

### Generation of ***T. turgidum–Ae. umbellulata*** F_1_hybrid seeds and identification of their chromosomes by in situ hybridization

A total of 30 *T. turgidum–Ae. umbellulata* triploid F_1_ plants were obtained. The seed-setting rate of the F_1_ plants among the 11 crosses ranged from 3.1% (STU 10) to 20.0% (STU 7), with a mean of 8.7% (Table 1). The germination rates of these F_1_ hybrid seeds ranged from 33.3% (STU 12 and STU 16) to 100.0% (STU 2, STU 8-STU 11, and STU 14) (Table 1). The chromosome numbers in the root tip cells of all the F_1_ hybrid plants were 2*n* = 3*x* = 21, and fluorescence in situ hybridization (FISH) and genomic in situ hybridization (GISH) verified that each root tip cell had a complete set of A, B, and U chromosomes from the diploid *Ae. umbellulata* and *T. turgidum* parents (Figure S1).

**Table 1:** Chromosome configuration at metaphase I and selfed seed-set rate of F_1_ hybrid plants

Cross	Triploid F_1_			CN in PMCs of F_1_	Observed no. of PMCs	Chromosome configuration				No. of chiasmata	Seed-set rate (%)	Mechanisms of unreduced gametes
	Seed-set rate (%)	No. of plants	Germination rate (%)			Univalent	Bivalent (II)		Trivalent (III)			
							Rod II	Ring II				
STU2 (Langdon/PI 554395)	3.3 (1/30) ^a^	1	100.0	21	150	15.42 ± 0.65 d	2.75 ± 0.27 ab	0	0.02 ± 0.02 b	2.79 b (0–5)	0.5 (2/400) ^a^	FDR & SDM
STU7 (Langdon/PI 428569)	20.0 (10/50)	8	80.0	21	150	16.96 ± 0.40 c	1.93 ± 0.19 c	0.01 ± 0.03 b	0.05 ± 0.03 ab	2.05 c (0–4)	0.8 (39/4840)	FDR & SDM
STU8 (Langdon/CIae 29)	3.6 (1/28)	1	100.0	21	150	17.81 ± 0.47 bc	1.40 ± 0.24 d	0.06 ± 0.05 a	0.09 ± 0.01 a	1.71 cd (0–4)	0.7 (7/1030)	FDR
STU9 (Langdon/PI 227436)	6.7 (2/30)	2	100.0	21	150	17.95 ± 0.41 b	1.49 ± 0.22 cd	0	0.02 ± 0.02 b	1.53 d (0–4)	5.0 (26/516)	FDR
STU10 (Langdon/PI 542365)	3.1 (2/64)	2	100.0	21	150	16.11 ± 0.57 d	2.40 ± 0.31 b	0.01 ± 0.01 b	0.03 ± 0.01 b	2.47 b (0–4)	1.8 (6/332)	FDR
STU11 (Langdon/PI 542376)	7.5 (3/40)	3	100.0	21	150	17.72 ± 0.35 bc	1.64 ± 0.17 cd	0	0	1.64 cd (0–4)	10.6 (149/1408)	FDR & SDM
STU12 (Langdon/PI 542377)	10.0 (3/30)	1	33.3	21	150	17.62 ± 0.12 bc	1.66 ± 0.10 cd	0	0.02 ± 0.03 b	1.70 cd (0–4)	11.4 (128/1124)	FDR
STU13 (Langdon/PI 542379)	16.7 (5/30)	4	80.0	21	150	17.63 ± 0.12 bc	1.67 ± 0.09 cd	0	0.01 ± 0.02 b	1.69 cd (0–5)	7.9 (78/994)	FDR & SDM
STU14 (Langdon/PI 542383)	15.6 (5/32)	5	100.0	21	150	19.71 ± 0.11 a	0.59 ± 0.04 e	0	0.03 ± 0.01 b	0.66 e (0–3)	13.0 (406/3128)	FDR & SDM
STU15 (PI 94668/PI 542364)	4.7 (3/64)	2	66.7	21	150	17.13 ± 0.25 bc	1.87 ± 0.11 cd	0	0.04 ± 0.02 b	1.95 cd (0–5)	0.5 (10/1856)	FDR
STU16 (PI 94668/PI 542365)	4.4 (3/68)	1	33.3	21	150	14.47 ± 1.03 e	3.17 ± 0.61 a	0.02 ± 0.02 b	0.04 ± 0.05 ab	3.31 a (0–6)	0 (0/930)	FDR

Note: The different lowercase letters in the same column indicate significant difference at *P* < 0.05. In the number of chiasmata column, the data were shown as mean and range. CN represents chromosome number. a. Number of seeds to the number of florets pollinated

### Meiosis in the ***T. turgidum–Ae. umbellulata*** F_1 _hybrids

**Fig. 1:**
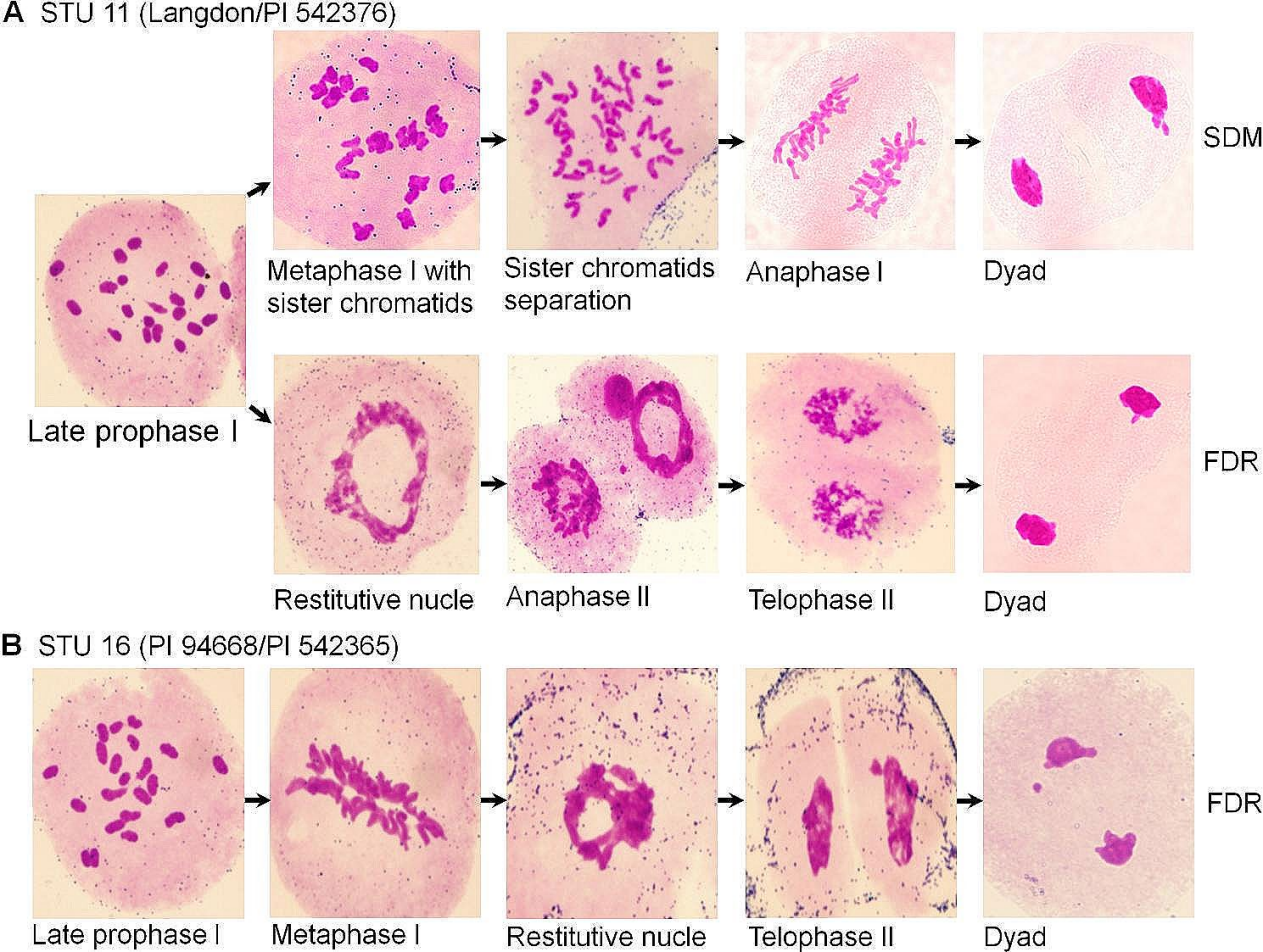
Mechanisms of unreduced gametes in the pollen mother cells (PMCs) of *Triticum turgidum*–*Aegilops umbellulata* triploid F_1_ hybrids STU 11 (**A**) and STU 16 (**B**)

The unreduced gamete formation and chromosome pairing during meiosis at the booting stage in 30 *T. turgidum–Ae. umbellulata* triploid F_1_ plants were investigated (Table 1). All 11 triploid F_1_ hybrid crosses could produce unreduced gametes, either only by FDR or by both FDR and SDM (Table 1). Five crosses (STU 2, STU 7, STU 11, STU 13, and STU 14) produced gametes by both FDR and SDM (Fig. 1A, Figure S2A–D and I–L), and the remaining six combinations (STU 8–STU 10, STU 12, and STU 15 and STU 16) only produced gametes via FDR (Fig. 1B, Figure S2E–H, and M–N). Additionally, some chromosome*-*specific behaviors during meiosis in the pollen mother cells (PMCs) of the 11 F_1_ hybrid crosses were also observed, such as lagging chromosomes (Figure S3C–E), chromosome bridges Figure S3F), micronuclei (Figure S3G), and multipolar division (Figure S3H).

**Fig. 2:**
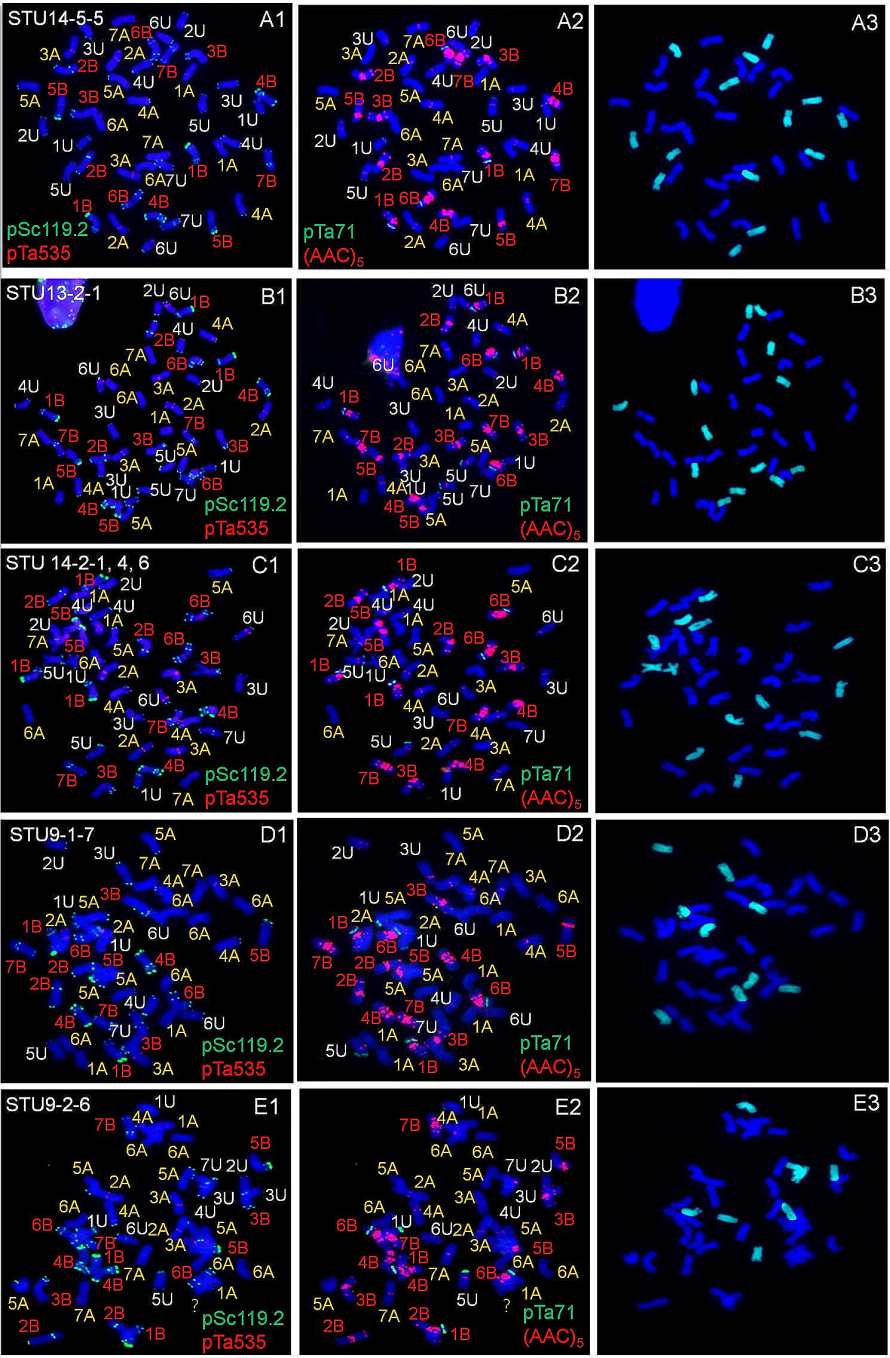
Fluorescence in situ hybridization (FISH) (**A1–E1**; **A2–E2**) and genome in situ hybridization (GISH) (**A3–E3**) of *Triticum turgidum*–*Aegilops umbellulata* euploids and pseudoeuploids. A1–A3. Euploids; B1–E3. Pseudoeuploids (B1–C3. 1B trisomic and 7U monosomic; D1–D3. 2U, 3U, 4U, 5U, 7U monosomic, 5 A tetrasomic, and 6 A pentasomic; E1–E3. 2 U, 3 U, 4 U, 5 U, 6 U, 7 U monosomic, 5 A tetrasomic, 6 A pentasomic, and one unknown)

The chromosome configurations at metaphase MI in the triploid F_1_ hybrids were analyzed (Table 1). The chromosome numbers in the PMCs of these F_1_ hybrid plants were 21. The number of univalent, rod bivalents (II), ring bivalents (II), and trivalents (III) (Figure S3A, B) among the 11 crosses ranged from 14.47 ± 1.03 to 19.71 ± 0.11, 0.59 ± 0.04 to 3.17 ± 0.61, 0 to 0.06 ± 0.05, and 0 to 0.09 ± 0.01, respectively. STU 8 had a significantly higher number of ring bivalents than the other 10 combinations, whereas STU 14 had significantly higher (*P* < 0.05) univalent (STU 14 vs. other crosses: > 19.5 vs. < 18.0) and lower (*P* < 0.05) rod bivalents (STU 14 vs. other crosses: < 0.6 vs. > 1.4) than the other 10 crosses. STU 16 had the highest number of chromosome chiasmata and rod bivalents among the 11 F_1_ hybrid crosses (Table 1).

The *T. turgidum–Ae. umbellulata* triploid F_1_ hybrid plants produced 0–128 seeds by selfing, which may be due to the formation of unreduced gametes, resulting in doubling success and fruiting of triploid F_1_ plants. The selfed seed-set rate of the 11 hybrid crosses ranged from 0 (STU 16) to 13.0% (STU 14), with a mean of 4.7%. The selfed seed-set rates of the *T. turgidum–Ae. umbellulata* F_1_ hybrid plants were significantly positively correlated with the number of univalents (*r* = 0.71^*^, *P* < 0.05) and significantly negatively associated with the number of rod II (*r* = − 0.37^*^, *P* < 0.05) and chiasmata (*r* = − 0.73^*^, *P* < 0.05).

### Chromosome numbers and compositions in the selfed seeds from triploid F_1_ hybrid plants

The chromosome numbers in the root tip cells of the selfed F_2_ seeds from the plants of 10 triploid F_1_ hybrid crosses (except for STU 16 for which there were no F_2_ seeds) were studied (Table 2). The chromosome numbers of most F_2_ plants in the 10 crosses ranged from 39 to 43, except for the two F_2_ plants of STU 9, which had 35 chromosomes. Among the 127 F_2_ plants from 10 crosses, the chromosome numbers 2*n* = 42 had the highest frequency of 54.3%, followed by 2*n* = 41 (30.7%), and the other chromosome numbers were less than 10%. Of the 10 crosses, the F_2_ seeds from STU 2 and STU 15 were all 2*n* = 42, which may be due to the limited sample numbers. In addition, two (STU 11 and STU 14), four (STU 7, STU 8, STU 9, and STU 10), and two crosses (STU 12 and STU 13) had the frequency of 2*n* = 42 chromosomes higher than, lower than, and equal to that of the non-42 chromosomes, respectively.

**Table 2:** Distribution of chromosome numbers in the F_2_ of *T. turgidum-Ae. umbellulata* hybrid plants in root tip cells

Cross	No. of plants with GCN (ratios of plants with GCN to total, %)						Total plants
	2n = 35	2n = 39	2n = 40	2n = 41	2n = 42	2n = 43	
STU2	0 (0)	0 (0)	0 (0)	0 (0)	2 (100)	0 (0)	2
STU7	0 (0)	1 (4.5)	6 (27.3)	10 (45.5)	5 (22.7)	0 (0)	22
STU8	0 (0)	0 (0)	0 (0)	3 (60.0)	2 (40.0)	0 (0)	5
STU9	2 (15.4)	0 (0)	1 (7.7)	3 (23.1)	6 (46.1)	1 (7.7)	13
STU10	0 (0)	0 (0)	1 (33.3)	1 (33.3)	1 (33.3)	0 (0)	3
STU11	0 (0)	1 (6.7)	0 (0)	5 (33.3)	9 (60.0)	0 (0)	15
STU 12	0 (0)	0 (0)	0 (0)	4 (50.0)	4 (50.0)	0 (0)	8
STU13	0 (0)	2 (9.1)	1 (4.5)	7 (31.8)	11 (50.0)	1 (4.5)	22
STU14	0 (0)	0 (0)	2 (5.7)	6 (17.1)	27 (77.1)	0 (0)	35
STU15	0 (0)	0 (0)	0 (0)	0 (0)	2 (100.0)	0 (0)	2
Total plants (ratios of plants with GCN to total, %)	2 (1.6)	4 (3.1)	11 (8.7)	39 (30.7)	69 (54.3)	2 (1.6)	127

GCN: given chromosome numbers

The chromosome compositions of the F_2_ seeds from the selfed F_1_ hybrid plants were investigated by FISH and GISH (Table S1). The results showed that the frequency of euploid *T. turgidum–Ae. umbellulata* (2*n* = 42, had three sets of complete A, B, and U chromosomes) (Fig. 2A1–A3) was 49.61% in comparison with 50.39% for aneuploids (2*n* ≠ 42) and pseudoeuploids (2*n* = 42 but some chromosomes lost or gained) (Figure S4). According to the composition of the A, B, and U chromosomes, the pseudoeuploids could be divided into three types (I, II, and III). All three pseudoeuploids lost some U chromosomes but gained some A or B chromosomes. Type I gained one 1 B chromosome but lost one 7 U chromosome (Fig. 2B1–C3), whereas type II and type III gained two 5 A and three 6 A chromosomes but differed in the absence (Fig. 2D1–D3) and presence of unknown chromosomes (Fig. 2E1–E3). Both the types II and III lost one U chromosome each for 2 U, 3 U, 4 U, 5 U, and 7 U but type III also lost one 6U chromosome.

The chromosome composition of aneuploids included 13 types: the loss of some A chromosomes but not gain (Figure S5 A1–B3) or gain of some unknown chromosomes (Figure S5 C1–C3), the loss of some B chromosomes but not gain (Figure S5 D1–E3) or gain of some unknown chromosomes (Figure S5 F1–F3), the loss of some U chromosomes (Figure S5 G1–I3), the simultaneous loss of some A and U chromosomes but not gain (Figure S5 J1*–*K3) or gain of some other A chromosomes (Figure S5 L1*–*L3), the simultaneous loss of some B and U chromosomes but not gain (Figure S5 M1–N3) or gain of some other B chromosomes (Figure S5 O1–O3), the simultaneous loss of some A and B chromosomes but not gain (Figure S5 P1–P3) or gain of some A chromosomes (Figure S5 Q1–Q3), the simultaneous loss of some A, B, and U chromosomes but gain of some other A and U chromosomes (Figure S5 R1–R3), and gain of U chromosomes (Figure S5 S1–S3), respectively.

**Fig. 3:**
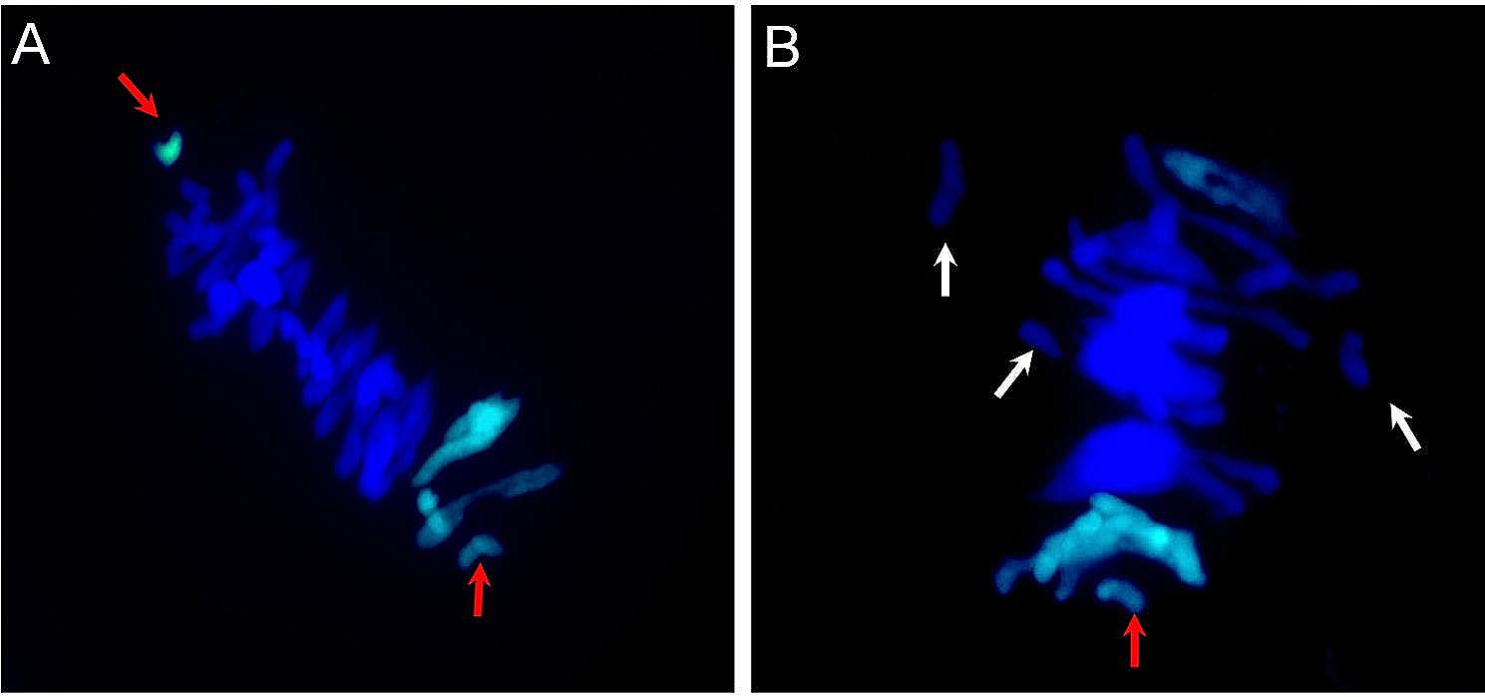
Genome in situ hybridization (GISH) of the pollen mother cells (PMCs) of STU 9 (Langdon/PI 227436) triploid F_1_ hybrids during meiosis. The DNA probe and block in the GISH figures were *Aegilops umbellulata* PI 227436 (green) and Langdon, respectively. The U and AB lagging chromosomes are indicated by red (**A**, **B**) and white arrows (**B**), respectively

According to FISH and GISH, chromosome deletions were detected in the A, B, and U chromosomes of the six crosses (STU 7–STU 9, STU 11, and STU 13–STU 14) (Table S1). To explain the chromosome deletions in these crosses, we made the chromsome observations for some crosses. As an example, meiosis in the PMCs of the STU 9 triploid hybrid F_1_ was observed. The results showed that lagging chromosomes including U (Fig. 3A, B) and A and B chromosomes (Fig. 3B) were found during meiosis. The lagging chromosomes were lost during meiosis, resulting in aneuploids. During meiosis, no association of the U chromosome with the A and B chromosomes was observed, which may be caused by the limited number of crosses and cells observed or perhaps there is no homoeologous relationship.

In the aneuploids of *T. turgidum–Ae. umbellulata*, the frequency of chromosome loss varied among genomes, with U being the highest (26.77%), followed by B (22.83%) and A (11.81%) (Figure S6A). The frequency of chromosome gain among the A, B, and U genomes was 3.94%, 3.94%, and 1.57%, respectively (Figure S6A). The frequency of chromosome loss also changed among chromosomes. Of the 21 chromosomes, 7 U had the highest frequency of chromosome loss (16.54%), followed by 1 B (9.45%), and the other five chromosomes (2 A, 7 A, 2 B, 4 B, and 5 B, each with 0.79%) had the lowest loss frequency (Figure S6B). Among the seven A chromosomes, 5 A had the highest loss frequency (3.94%), followed by 1 A, 3 A, and 6 A (each with 2.36%), and the loss frequency of the other three A chromosomes was lower than 2.00%. Among the seven B chromosomes, 1 B had the highest loss frequency (9.45%), followed by 3 B (7.09%), and the loss frequency of the other five B chromosomes was less than 3%. Among the seven U chromosomes, 7 U had the highest loss frequency (16.54%), followed by 3 U and 4 U (7.87%), and the loss frequency of the other four U chromosomes was less than 6.00%. The frequency of chromosome gain also varied among chromosomes. Among the 21 chromosomes, only seven chromosomes, namely, 1 A, 3 A, 5 A, 6 A, 1 B, 1 U, and 6 U, were gained in aneuploids. Of these, 1 B had the highest gain frequency (3.94%), followed by 6 A (2.36%), and the gain frequency of the other five chromosomes was less than 2.00% (Figure S6B). The chromosome loss/gain in the A, B, and U chromosomes varied among crosses (Table S1). Three crosses each showed no loss/gain in chromosomes A and U (STU 2, STU 12, and STU 15) and B (STU 2, STU 10, and STU 15). STU 13 (1 A, 3 A, 4 A, 5 A, and 6 A), STU 7 (1 B, 2 B, 3 B, and 7 B) and STU14 (1 B, 3 B, 6 B, and 7 B), and STU 9 (1–7 U) were the crosses with more chromosome loss/gain in the A, B, and U genomes, respectively.

**Fig. 4:**
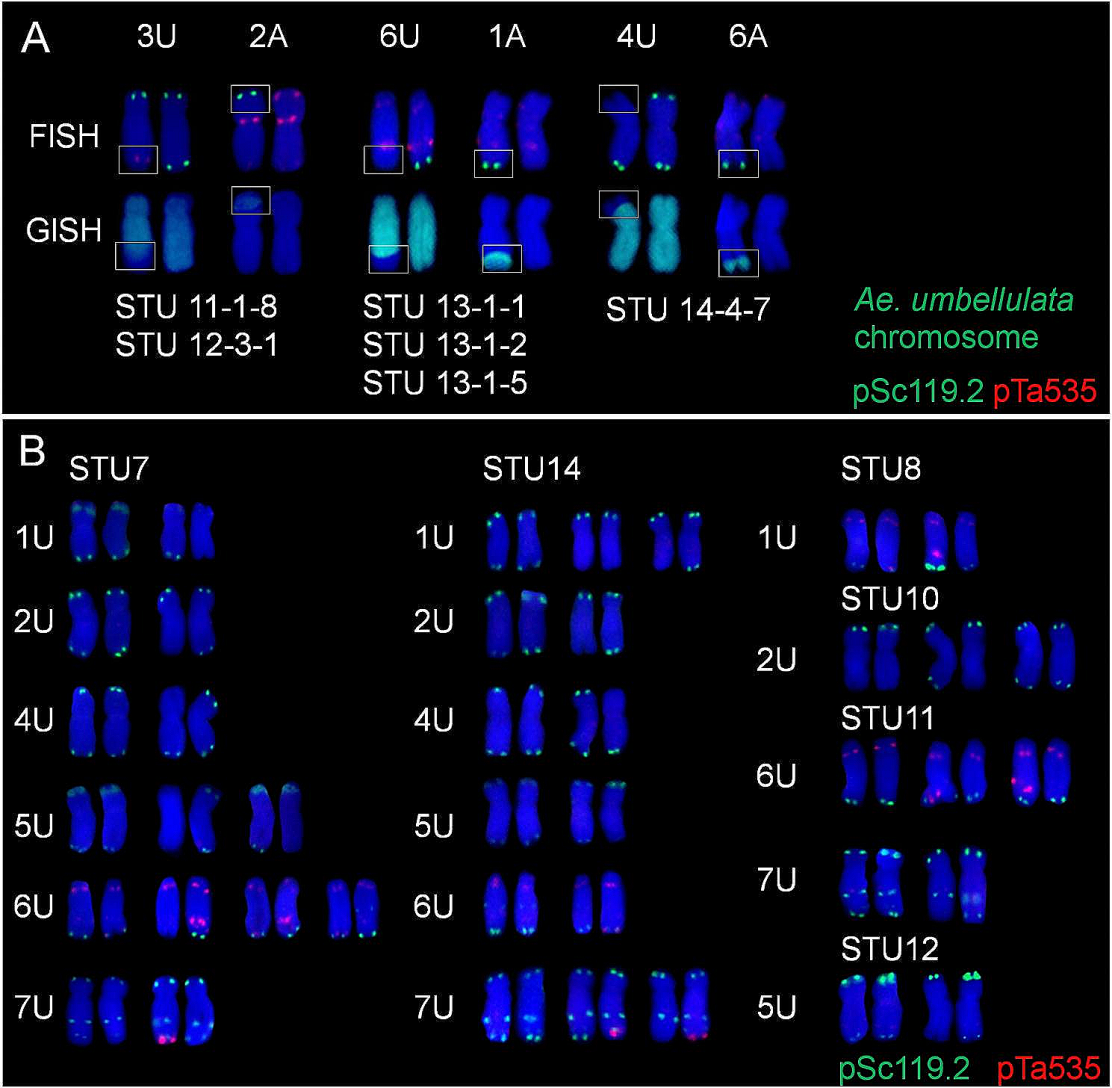
Translocation types (**A**) and polymorphic U chromosomes (**B**) in *Triticum turgidum*–*Aegilops umbellulata* amphidiploids

In addition to chromosome loss/gain, the selfed F_2_ seeds from the triploid hybrid F_1_ plants of *T. turgidum–Ae. umbellulata* were involved in three types of chromosome translocations between U and A chromosomes (Fig. 4A), which included 3UL·2AS (STU 11*-*1*-*8 and STU 12*-*3*-*1), 6UL·1AL (STU 13*-*1*-*1, STU 13*-*1*-*2 and STU 13*-*1*-*5), and 4US·6AL (STU 14*-*4*-*7).

In addition to the variations in chromosome structure and numbers described above, polymorphic U chromosomes were also detected in the amphidiploids from different crosses (Fig. 4B). For example, STU 7 had two types of 1 U, 2 U, 4 U, and 7 U, three types of 5 U, and four types of 6 U. STU 14 had two types of 3 U, 4 U, 5 U, and 6 U and three types of 2 U and 7 U. Three crosses (STU 8, STU 10, and STU 12) showed polymorphism in only one U chromosome, involving 6 U (two types), 2 U (three types), and 5 U (two types), respectively. STU 11 had three and two polymorphic types in 6 U and 7 U, respectively. The remaining three crosses (STU 2, STU 9, and STU 15) showed no polymorphism in all U chromosomes.

## Discussion

### The role of unreduced gametes in wheat distant hybridization

Unreduced gametes have been discovered in many haploids of Triticeae [13, 25, 26], such as hexaploid/tetraploid wheat with rye, and they are an important means for the origin of Triticeae [27, 28]. It was estimated that the average frequency of unreduced gametes in hybrid was 50 times greater than non-hybrid populations [29] and approximately 0.1–2.0% of gametes in a non-hybrid plant population were expected to be unreduced [30]. In the F_1_ hybrids of tetraploid wheat with *Ae. tauschii*, *Ae. longissima*, and *Ae. comosa*, both FDR and SDM mechanisms are involved in unreduced gamete formation [9, 18, 21]. Only FDR was found in the F_1_ hybrids of wheat–*Ae. triuncialis*, as well as in some F_1_ hybrids of *T. turgidum*–*Ae. markgrafii* and *T. turgidum*–*Ae. tauschii* [14, 19, 21]. Homologous chromosome pairing can prevent the formation of unreduced gametes. For example, no unreduced gamete formation was found in the haploid hybrid F_1_ of wheat–*Ae. cylindrica* (ABDDC) and tetraploid wheat with tetraploid *Ae. tauschii* (2*n* = 4*x* = 28, DDDD) due to homologous pairing between the D genomes [20, 31]. The present results showed that unreduced gametes were produced in triploid F_1_ hybrids of tetraploid wheat ssp. *durum* var. Langdon and ssp. *dicoccon* PI 94668 with *Ae. umbellulata*, providing a theoretical basis for the formation of doubled haploids by the natural doubling of triploid *T. turgidum*–*Ae. umbellulata* hybrids. Both FDR and SDM pathways of unreduced gamete formation have been found in the triploid hybrids of Langdon with *Ae. comosa* [21]. In this study, only FDR and both FDR and SDM were found in the triploid F_1_ hybrids of durum wheat Langdon and *Ae. umbellulata*, but only FDR occurred in the two triploid F_1_ hybrids of PI 94668 and *Ae. umbellulata*, which may be due to the limited number of crosses that were used. In the FDR, no homologous chromosomes were paired (univalent formation) and separated during meiosis I or occurred at a very low frequency, whereas the two sister chromatids of homologous chromosomes moved to opposite poles during the second division [11]. Interspecific hybridization is an alternative and prospective strategy to introduce valuable traits from wild relative species into new cultivars but usually produce mostly sterile offspring due to the barriers of chromosome pairing during meiosis [12]. The FDR gamete formation had the advantage of transferring parental heterozygosity and maintain epistatic interactions other than cross-over fragments, and the formation of the 2n-gametes can be used to develop sexual polyploids with more genetic vigor, better yield and more resistant to biotic and abiotic stresses [32].

The selfed seed-set rate of F_1_ hybrid plants is an important index for measuring the frequency of unreduced gamete formation [9, 31, 33]. During the meiosis of PMCs of tetraploid wheat*-Ae. tauschii* hybrids, the ratio of dyads and the frequency of unreduced gametes are positively correlated with the seed-setting rate of selfed F_1_ hybrid plants [14]. Compared with the selfed seed-set rate of F_1_ hybrid plants of tetraploid wheat–*Ae. tauschii* (mean: 5.83%, range: 0*–*18.57%) [12] and *T. turgidum*–*Ae. comosa* (mean: 5.44%, range: 1.96–11.02%) [21], the selfed seed-set rate of the present *T. turgidum*–*Ae. umbellulata* F_1_ hybrid plants (mean: 4.7%, range: 0*–*13.0%) was lower than both [12, 21], although it was higher than that of the F_1_ hybrid plants of *T. turgidum*–*Ae. umbellulata* in our previous report (mean: 1.22%, range: 0.09*–*4.65%) [7]. The low seed-setting rate of these F_1_ hybrids may be ascribed to the lower number of univalents (14.47*–*19.71 vs. 20.26*–*20.87) and higher number of Rod II bivalents (0.59*–*3.17 vs. 0.05*–*0.34) and chiasmata (0.66*–*3.31 vs. 0.07*–*0.38) compared with those of the *T. turgidum*–*Ae. comosa* F_1_ hybrids [21]. However, the higher seed-setting rate of the present F_1_ hybrids of *T. turgidum*–*Ae. umbellulata* compared with that of *T. turgidum*–*Ae. markgrafii* F_1_ hybrids could be explained by a higher number of univalents and a lower number of Rod II bivalents and chiasmata in the *T. turgidum*–*Ae. umbellulata* hybrids [21]. The low frequency of unreduced gamete formation with fewer univalents, leading to a low seed-setting rate, was also detected in *T. durum*–*Ae. longissima* hybrids [18, 28].

### Chromosome variation of F_1_ hybrids and their offsprings of wheat and related species

Polyploid plants are highly tolerant to aneuploidy in comparsion with the diploid species. In most allopolyploid plants, homologous chromosome mismatch and the formation of multivalents are the main causes of aneuploidy and chromosome structure variation [34–36]. Theoretically, the *Ph1* gene can promote homologous chromosome pairing and inhibit homoeologous chromosome pairing between common wheat and synthetic wheat, and the meiosis of their offspring should be very stable, synthetic wheat and its offspring have a high frequency of chromosome number variation [14, 37]. Chromosome number variation is the main cause of aneuploids, which is common in nascent synthetic wheat and even in higher generations, and the frequency changes greatly among generations, ranging from 20 to 100% [37]. The newly synthetic wheat containing the active *Ph1* gene was also associated with meiotic abnormality and aneuploidy in a parent-dependent manner [38]. The chromosome numbers in synthetic wheat range from 39 to 45, and the frequency of euploids (2*n* = 42) is 51.4% [39]. The chromosome numbers (35–43) and the frequency of euploids (49.61%) in the current nascent synthetic *T. turgidum*–*Ae. umbellulata* species are close to those of synthetic hexaploid wheat [39]. The frequency of aneuploids in synthetic wheat SHW*-*L1 was 48.6%, though it decreased to 12.5% in a recombinant inbred line containing SHW*-*L1 blood [39] and was reduced to 1.3% and 3.0%, respectively, in the new wheat varieties ‘Shumai 969’ and ‘Shumai 830’ derived from SHW*-*L1 [40]. These results suggest that hybridization and multiple generations of self-crossing could significantly enhance the chromosome stability of synthetic wheat species, including *T. turgidum*–*Ae. umbellulata* and its offspring. In addition to variations in chromosome number, chromosome structure variation, such as translocation, duplication, deletion, and inversion, has also been discovered in synthetic wheat [41, 42]. In the selfed F_2_ seeds from triploid hybrid plants of *T. turgidum*–*Ae. umbellulata*, we observed a wide range of chromosome number variation and three types of chromosome translocations involving 3 U and 2 A, 6 U and 1 A, and 4 U and 6 A, as well as different types of polymorphic U chromosomes (Fig. 4A). These results suggested that alterations in chromosome number and structure variation still exist in the newly synthetic wheat species *T. turgidum*–*Ae. umbellulata*. A high frequency of aneuploid plants is an indicatior of chromosomal instability. In the primary synthetic wheat, wide variation of chromosome numbers between plants was colsely related to the frequency of univalent during meiosis [39]. Univalents separate irregularly during meiosis and their derivative chromosomes usually not equally delivered to offspring nuclei or lost in the formation of micronuclei [39]. Univalents in wheat subject to chromosome breakage and generate chromosome fragments and the possibility of breakage-fusion causing translocation chromosomes [43].

The *Aegilops tauschii* D genome acts as a key genome in the wheat*–Aegilops* complex group. The phenotypic characteristics encoded by the D genome have hardly changed during evolution, whereas those of the coexisting genomes usually change significantly [44]. In the three genomes (A, B, and D) of synthetic wheat, the frequency of chromosome loss or gain is not completely consistent, with B being the highest, followed by A, with the last added D genome being the most stable. The frequency of chromosome loss and gain in each genome differs among chromosomes, which may be related to the self-stability of the D genome of *Ae. tauschii*, and once combined with other genomes by allopolyploidy, it might cause instability in other genomes [37]. In the synthetic *T. turgidum*–*Ae. umbellulata* species, the frequency of chromosome loss was the highest for the U genome, followed by the B genome, whereas the A genome was the most stable. To overcome the chromosome instability of synthetic *T. turgidum*–*Ae. umbellulata*, hybridization and multiple generations of self-crossing could be applied in order to retain less genomic content from the synthetic. As an example, the chromosome stability of nascent wheat was largely elevated by increasing the genetic background of common wheat through such way [39].

## Materials and methods

### Plant materials

A total of 30 *T. turgidum–Ae. umbellulata* triploid F_1_ hybrid plants from the 11 crosses, which were named STU 2 and STU 7–STU 16 (Table S2), were produced in our lab by *T. turgidum* (2n = 4x = 28, AABB) subsp. *durum* var. Langdon crossed with nine *Ae. umbellulata* accessions (2n = 2x = 14, UU) and *T. turgidum* subsp. *dicoccum* PI 94668 crossed with two *Ae. umbellulata* accessions. All the *Ae. umbellulata* accessions and *T. turgidum* ssp. *dicoccum* accession PI 94668 were kindly supplied by USDA-AGRS germplasm bank (https://www.ars-grin.gov/). The voucher specimens for *Ae. umbellulata* (Deposition number 201,400,199) and *T. turgidum* ssp. *dicoccum* (201,404,987) that were identified by Prof. Yang Junliang in our Institute and that of *T. turgidum* ssp. *durum* (NAS00533826) was idenfied by Prof. Guo Benzhao in Northwest Plateau Biology Institute, Chinese Academy of Sciences can be found at Chinese virtual herbaium (https://www.cvh.ac.cn/).

### Production of triploid F_1_ hybrids

Eleven triploid F_1_ hybrids were produced using *T. turgidum* ssp. as the female parent and *Ae. umbellulata* as the male parent following reference [45]. During the generation of F_1_ hybrid seeds, no embryo rescue or hormone treatment was applied. The F_1_ hybrid seeds were germinated in petri dishes, and the chromosome numbers in the root tip cells were cytologically checked to retain those with 21 chromosomes. Triploid plants with 2*n* = 3*x* = 21 (Figure S1) were transplanted into the field of Wenjiang experimental farm of Chengdu in Sichuan, China for further analysis. No chemical reagent was used for doubling the chromosomes of these triploid F_1_ plants, and therefore, their chromosomes were doubled via spontaneous unreduced gametes.

### Cytological observations

Chromosome numbers in the root tip cells and PMCs at meiotic metaphase I were determined following reference [9]. A total of 150 cells were observed in each plant of every cross. The chromosome configurations including the number of univalent (I), ring and rod bivalents (II), trivalents (III), and quadrivalents (IV) were recorded at the metaphase of the first meiosis. The number of chimastata for the univalent, rod bivalent, and ring bivalent and trivalent was calcaulated as 0, 1, and 2, respectively. The chromosome numbers of 30 F_1_ triploid plants and the selfed seeds (eight seeds were randomly selected for those crosses generate more than eight seeds, and all for those produce less than five seeds) were determined. All the triploid hybrid seeds from the 11 crosses and one seed each from the selfed seeds of each F_1_ plant were analyzed for chromosome constitution by in situ hybridization. Slides were prepared for FISH and GISH [46, 47]. Four oligonucleotide probes, namely, Oligo*-*pTa*-*535 (pTa535), Oligo*-*pSc119.2 (pSC119.2), Oligo*-*pTa71 (pTa71) [48], and (AAC)_5_ [49], were labeled by 6*-*carboxyfluorescein (6*-*FAM) or 6*-*carboxytetramethylrhodamine (Tamra) and synthesized by Sangon Biotech in Shanghai, China. These four probes could distinguish all the A, B, and U chromosomes in *T. turgidum–Ae. umbellulata* hybrids. The probe combinations pSc l19.2, (AAC)_5_, and pTa71 could distinguish all the U chromosomes, whereas the probes pSc119.2 and pTa535 could differentiate all the A and B chromosomes [7]. The genomic DNA used for GISH was isolated from the young leaves of the *Ae. umbellulata* accessions PI 227436 and PI 554395 and the tetraploid wheat lines Langdon and PI 94668 using a modified cetyltrimethylammonium bromide (CTAB) method [50]. The genomic DNA of *Ae. umbellulata* was used as a probe and labeled by nick translation with Chroma Tide Alexa Fluor 488*-*5*-*dUTP (Invitrogen, USA; no. C11397, green coloration). The genomic DNA of tetraploid wheats (Langdon, or PI 94668) was used as a blocker. The following steps, including hybridization, image capture and treatment, and re*-*hybridization, were the same as described by [51].

### Statistical analyses

The data were statistically analyzed using Excel 2019 (Microsoft Corp., Redmond, WA, USA) and SPSS 27 (IBM Corp., Armonk, NY, USA). One-way analysis of variance was performed for the number of chiasmata and chromosome configuration and the least significant difference was applied for Post-hoc test of significant difference. The chromosome signals in multi*-*channel were combined with DP Manager (Olympus, Tokyo, Japan), and chromosome extraction and image processing were carried out with Adobe Photoshop 12.0.3 (Adobe Systems Incorporated, San Jose, CA, USA).

## Conclusions

The unreduced gamete formation pathways in 30 triploid F_1_ hybrid plants of *T. turgidum–Ae. umbellulata* were studied herein. FDR alone and both FDR and SDM were found in different crosses. The chromosome numbers in the F_2_ plants derived from the selfed seeds of the triploid F_1_ hybrid plants varied from 35 to 43, and the frequencies of euploids and aneuploids in these F_2_ plants were 49.61% and 50.39%, respectively. In the aneuploid F_2_ plants, all 21 chromosomes and seven of the 21 chromosomes (1 A, 3 A, 5 A, 6 A, 1 B, 1 U, and 6 U) were involved in chromosome loss and gain, respectively. U had a higher frequency of chromosome loss (26.77%) and a lower frequency of chromosome gain than the B (loss/gain: 22.83%/3.94%) and A (loss/gain: 11.81%/3.94%) genomes, respectively. Furthermore, three types of chromosome translocations related to 3 U and 2 A, 6 U and 1 A, and 4 U and 6 A were also identified in the F_2_ plants. In addition to variations in chromosome number and structure, polymorphic FISH karyotypes were also found in the F_2_ plants for all the U chromosomes. These results are valuable for producing naturally occurring *T. turgidum*–*Ae. umbellulata* amphidiploids, which is a bridge material for distant hybridization between wheat and *Ae. umbellulata.*

### Electronic supplementary material

Below is the link to the electronic supplementary material.

Supplementary Material 1

Supplementary Material 2

## Data Availability

All data generated or analysed during this study are included in this published article and its supplementary information files.

## Abbreviations

FDR: First division restitution
FISH: Fluorescence in situ hybridization
GISH: Genomic in situ hybridization
PMCs: Pollen mother cells
QTLs: Quantitative trait loci
SDM: Single-division meiosis
