# Supplementary material for: Chromosome stability of synthetic Triticum turgidum–Aegilops umbellulata hybrids
Source: BMC Plant Biol. 2024 May 13;24:391. doi: 10.1186/s12870-024-05110-8 (PMC11089697; doi:10.1186/s12870-024-05110-8)
Supplement: Supplementary file 1 — Supplementary Material 1 [file 12870_2024_5110_MOESM1_ESM.docx]

**Table S1.** Chromosome composition of the selfed seeds from triploid F_1_ hybrid plants between *Triticum turgidum* and *Aegilops umbellulata*.

| Cross | Plant code | Chromosome number | Chromosome loss ^a^ | Chromosome gain ^a^ | Chromosome structure variation |
| --- | --- | --- | --- | --- | --- |
| STU 2 | 1-(1, 2) | 42 |  |  |  |
| STU 7 | 1-1 | 40 | 2A', 7U' |  | 6U' |
|  | 1-2 | 40 | 2U', 4U' |  |  |
|  | 2-1 | 40 | 2B', 7U' |  | 2U', 6U' |
|  | 2-(2, 4) | 41 | 7U' |  |  |
|  | 2-3 | 39 | 7B', 1U', 3U' |  | 4U', 6U', 7U' |
|  | 2-(5, 6, 8), 8-4 | 41 | 1B' |  | 6U', 7U' |
|  | 2-7 | 42 |  |  | 5U', 6U', 7U' |
|  | 3-1 | 41 | 2U' |  | 5U', 6U', 7U' |
|  | 4-1 | 41 | 2A' |  | 6U', 7U' |
|  | 5-1, 9-2 | 42 |  |  | 6U', 7U' |
|  | 5-2, 9-1 | 40 | 1B', 3B' |  | 6U', 7U' |
|  | 8-(3, 6) | 42 |  |  | 1U' |
|  | 8-5 | 41 | 7A |  | 7U' |
|  | 8-7 | 41 | 2B' |  | 6U', 7U' |
|  | 8-8 | 40 | 2B', 7U' |  | 2U', 5U' |
| STU 8 | 1-(1, 2) | 42 |  |  | 6U' |
|  | 1-4 | 41 | 3A',1U' | 1A' | 6U' |
|  | 1-5 | 41 | 3B' |  | 6U' |
|  | 1-7 | 41 | 1B', 4B' | X' |  |
| STU 9 | 1-(1, 4, 5), 2-8 | 42 |  |  |  |
|  | 1-6 | 40 | 3U', 4U' |  |  |
|  | 1-7 | 42 | 2U', 3U', 4U', 5U', 7U' | 5A'', 6A''' |  |
|  | 1-8 | 35 | 6A', 1U', 2U', 3U', 4U', 5U', 7U' |  |  |
|  | 2-1 | 41 | 3B', 7U' | 1B' |  |
|  | 2-2 | 41 | 3A' |  |  |
|  | 2-4 | 43 |  | 1U' |  |
|  | 2-5 | 41 | 7U' |  |  |
|  | 2-6 | 42 | 2U', 3U', 4U', 5U', 6U', 7U' | 5A'', 6A''', X' |  |
|  | 2-7 | 35 | 1U', 2U', 3U', 4U', 5U', 6U',7U' |  |  |
| STU 10 | 2-2 | 40 | 6A', 3U' |  | 2U'' |
|  | 2-3 | 41 | 2U' |  |  |
|  | 2-5 | 42 |  |  | 2U' |
| STU 11 | 1-(1, 3), 3-(3, 4, 5, 7, 8) | 42 |  |  |  |
|  | 1-(2,6) | 41 | 1B' |  |  |
|  | 1-(5,7) | 42 |  |  | 6U', 7U' |
|  | 1-8 | 41 | 6B' |  | T2AL•2AS-3UL' |
|  | 3-1 | 41 | 4U' |  |  |
|  | 3-2 | 39 | 4A', 1B', 2B' |  |  |
|  | 3-6 | 41 | 3U' |  | 6U'' |
| STU 12 | 3-1 | 41 | 6B' |  | T2AL•2AS-3UL' |
|  | 3-(2, 3, 5, 8) | 42 |  |  |  |
|  | 3-(4, 6) | 41 | 1B' |  |  |
|  | 3-7 | 41 | 1B' |  | 5U' |
| STU 13 | 1-(1, 2, 5) | 42 |  |  | T1AS•1AL-6UL' |
|  | 1-6 | 40 | 1A', 5A' |  |  |
|  | 1-7, 4-1 | 41 | 7U' |  |  |
|  | 2-1 | 42 | 7U' | 1B' |  |
|  | 2-(2, 7, 8), 3-(4, 5), 4-(2, 8) | 42 |  |  |  |
|  | 2-3 | 39 | 4A', 3B', 5B', 7B' | 6A' |  |
|  | 2-6, 4-3 | 41 | 3B' |  |  |
|  | 3-3 | 43 | 3A' | X', X' |  |
|  | 3-6 | 41 | 1A' |  |  |
|  | 4-5 | 41 | 4U' |  |  |
|  | 4-6 | 39 | 3B'', 6U' |  |  |
|  | 4-7 | 41 | 6U' |  |  |
| STU 14 | 1-(1-6), 3-1, 5-(2, 8) | 42 |  |  |  |
|  | 1-7 | 40 | 7B', 4U' |  | 3U' |
|  | 1-8 | 42 |  |  | 3U' |
|  | 2-1 | 42 | 7U' | 1B' | 4U' |
|  | 2-2, 3-7, 4-8, 5-(4-7) | 42 |  |  | 2U' |
|  | 2-3 | 42 |  |  | 2U'' |
|  | 2-(4, 6) | 42 | 7U' | 1B' | 5U' |
|  | 2-5 | 41 | 1A', 6B', 3U' | 3A', 6U' | 4U' |
|  | 2-7 | 41 | 4U' |  |  |
|  | 2-8 | 41 | 1B' |  |  |
|  | 3-2, 4-5 | 42 |  |  | 4U' |
|  | 3-6 | 41 | 5A' |  | 7U' |
|  | 3-8, 4-(3, 4) | 42 |  |  | 6U', 7U' |
|  | 4-1 | 40 | 6A', 3U' |  | 6U', 7U' |
|  | 4-6 | 42 |  |  | 5U' |
|  | 4-7 | 41 | 3B' |  | T6AS•6AL-4US' |
|  | 5-1 | 41 | 7U' |  |  |
| STU 15 | 2-(1, 7) | 42 |  |  |  |

Note: a indicates chromosome loss or gain compared to the euploid. 2A' represents one 2A chromosome. 3B'' represents two 3B chromosomes. X represents an unknown chromosome.

**Table S2.** Materials used in this experiment.

| Material | Genome | No. of chromosome | Accessions (cross) |
| --- | --- | --- | --- |
| *Ae. umbellulata* | UU | 14 | PI 227436 PI 428569 PI 542364 PI 542365 PI 542376  PI 542377 PI 542379 PI 542383 PI 554395 CIae 29 |
| *T. turgidum* ssp. *dicoccum*  *T. turgidum* ssp. *durum* | AABB | 28 | PI 94668  Langdon |
| Triploid *T. turgidum-Ae. umbellulata* F_1_ hybrids | ABU | 21 | STU 2 (Langdon/PI 554395) STU 7 (Langdon/PI 428569)  STU 8 (Langdon/CIae29) STU 9 (Langdon/PI 227436)  STU 10 (Langdon/PI 542365) STU 11 (Langdon/PI 542376)  STU 12 (Langdon/PI 542377) STU 13 (Langdon/PI 542379)  STU 14 (Langdon/PI 542383) STU 15 (PI 94668/PI 542364)  STU 16 (PI 94668/PI 542365) |
